# Evaluation of Borage Extracts As Potential Biostimulant Using a Phenomic, Agronomic, Physiological, and Biochemical Approach

**DOI:** 10.3389/fpls.2017.00935

**Published:** 2017-06-07

**Authors:** Roberta Bulgari, Silvia Morgutti, Giacomo Cocetta, Noemi Negrini, Stefano Farris, Aldo Calcante, Anna Spinardi, Enrico Ferrari, Ilaria Mignani, Roberto Oberti, Antonio Ferrante

**Affiliations:** ^1^Department of Agricultural and Environmental Sciences – Production, Landscape, Agroenergy, Università degli Studi di MilanoMilan, Italy; ^2^Department of Food, Environmental and Nutritional SciencesUniversità degli Studi di Milano, Milan, Italy

**Keywords:** *Borago officinalis* L., image analysis, *Lactuca sativa* L., non-destructive measurements, phenols, photosynthesis

## Abstract

Biostimulants are substances able to improve water and nutrient use efficiency and counteract stress factors by enhancing primary and secondary metabolism. Premise of the work was to exploit raw extracts from leaves (LE) or flowers (FE) of *Borago officinalis* L., to enhance yield and quality of *Lactuca sativa* ‘Longifolia,’ and to set up a protocol to assess their effects. To this aim, an integrated study on agronomic, physiological and biochemical aspects, including also a phenomic approach, has been adopted. Extracts were diluted to 1 or 10 mL L^–1^, sprayed onto lettuce plants at the middle of the growing cycle and 1 day before harvest. Control plants were treated with water. Non-destructive analyses were conducted to assess the effect of extracts on biomass with an innovative imaging technique, and on leaf photosynthetic efficiency (chlorophyll *a* fluorescence and leaf gas exchanges). At harvest, the levels of ethylene, photosynthetic pigments, nitrate, and primary (sucrose and total sugars) and secondary (total phenols and flavonoids) metabolites, including the activity and levels of phenylalanine ammonia lyase (PAL) were assessed. Moreover, a preliminary study of the effects during postharvest was performed. Borage extracts enhanced the primary metabolism by increasing leaf pigments and photosynthetic activity. Plant fresh weight increased upon treatments with 10 mL L^–1^ doses, as correctly estimated by multi-view angles images. Chlorophyll *a* fluorescence data showed that FEs were able to increase the number of active reaction centers per cross section; a similar trend was observed for the performance index. Ethylene was three-fold lower in FEs treatments. Nitrate and sugar levels did not change in response to the different treatments. Total flavonoids and phenols, as well as the total protein levels, the *in vitro* PAL specific activity, and the levels of PAL-like polypeptides were increased by all borage extracts, with particular regard to FEs. FEs also proved efficient in preventing degradation and inducing an increase in photosynthetic pigments during storage. In conclusion, borage extracts, with particular regard to the flower ones, appear to indeed exert biostimulant effects on lettuce; future work will be required to further investigate on their efficacy in different conditions and/or species.

## Introduction

In the last years, the use of biostimulants has been constantly increasing for sustainable agriculture, because these substances enhance nutrient use efficiency, reduce fertilizers consumption, stimulate plant development and growth (Kunicki et al., 2010; Calvo et al., 2014; Halpern et al., 2015; Le Mire et al., 2016), and counteract stress factors, eventually enhancing crop quality and yield (Ziosi et al., 2013; Van Oosten et al., 2017). The interest in this sector is evidenced by the significant increase of research papers focused on it and by its economical relevance. The market of biostimulant products is projected to increase by 12% annually (Calvo et al., 2014), reaching $2,524.02 million by 2019 (Povero et al., 2016). Biostimulants are generally made of raw organic materials containing bioactive compounds. Their nature is heterogeneous, since they include humic acids, protein hydrolysates, plant growth-promoting *Rhizobacteria* and fungi, and extracts from seaweeds and higher plant species (Ertani et al., 2013, 2016; du Jardin, 2015). For this reason, also their chemical composition is highly heterogeneous, including mineral elements, vitamins, amino acids, chitin, chitosan, and poly- and oligosaccharides, and therefore it is partly unknown. Moreover, their chemical complexity and the wide range of molecules present make very difficult to understand which are the most active compounds (Brown and Saa, 2015; Bulgari et al., 2015; du Jardin, 2015; Posmyk and Szafrańska, 2016; Yakhin et al., 2017).

Under a biochemical point of view, the increased plant growth induced by biostimulants can be associated with an increase in amino acid levels and enhanced protein biosynthesis, as well as in carbohydrate concentration in leaves (Abdalla, 2013). Higher sugar levels in plants treated with biostimulants have been found in several species, associated with higher chlorophyll accumulation, net photosynthesis (Abbas and Akladious, 2013; Abdalla, 2013), and quantum efficiency of photosystem II (Ferrini and Nicese, 2002; Amanda et al., 2009; Ertani et al., 2012). In lettuce, biostimulant application at the nursery level positively affects plant growth by increasing the development of shoots and roots, strongly stimulating root growth and increasing leaf area (Vernieri et al., 2002; Amanda et al., 2009). Moreover, a positive role on yield and quality of head lettuce has been reported, with particular regard to the reduction of nitrate that is an important issue for human health (Shehata et al., 2016).

Biostimulants are capable to counteract plant stresses that are usually related to a burst in ethylene synthesis and sensitivity, and eventually affect produce quality by altering or accelerating tissue senescence (Saltveit, 1999). The activation of stress responses in plants is often accompanied by the synthesis of secondary metabolites (Mazid et al., 2011; Ramakrishna and Ravishankar, 2011) that often act as antioxidants scavenging *in vivo* and *in vitro* (Cook and Samman, 1996) the free radicals (Halliwell, 2008) produced in stress-induced oxidative reactions (Sharma et al., 2012), and counteracting the free radical-induced damages to cell components. In animal systems, and particularly in humans, several studies have shown that a diet rich in antioxidants from plant-derived foods may prevent the onset of a wide range of chronic-degenerative diseases (Manach et al., 2004; Vauzour et al., 2010; Martin et al., 2013; Bertoia et al., 2016).

The largest group of bioactive beneficial secondary plant metabolites is represented by phenolic compounds, ubiquitous in all tissues of higher plants, where they play an important role providing the plant with specific adaptations to changing environmental conditions and eliciting defense mechanisms (Caretto et al., 2015 and references therein). Phenolic compounds are synthesized in the phenylpropanoid pathway, that produces an array of different substances including, amongst others, phenolic acids and flavonoids (El Gharras, 2009), reported to possess powerful antioxidant activity *in vitro* (Kähkönen et al., 1999). The first committed enzyme in the phenylpropanoid biosynthetic pathway is phenylalanine ammonia lyase (PAL; E.C. 4.3.1.5), that catalyzes the non-oxidative deamination of phenylalanine to *trans*-cinnamic acid (CA), common substrate for the biosynthesis of various phenylpropanoid compounds (Ferrer et al., 2008), a step that represents a crucial link between primary and secondary metabolism. PAL activity is positively related with increased production of phenylpropanoids (Vogt, 2010). Vegetal extracts derived from red grape, blueberry fruits and hawthorn leaves or from oak, affect PAL activity and expression of *PAL* genes as well as the levels of polyphenols in maize or grape, respectively, when applied as biostimulant (Ertani et al., 2011, 2016; Pardo-García et al., 2014). To our knowledge, studies on PAL in lettuce have dealt mainly with its involvement in postharvest disorders (Ke and Saltveit, 1986, 1989), tissue browning (Campos et al., 2004), and pigment biosynthesis under different temperature regimes (Chon et al., 2012). In the last decade, the availability of relatively inexpensive and high-performance optical systems, digital cameras and associated software technologies has boosted the development of phenotyping systems, i.e., of semi-automatic or automatic devices enabling repeated and non-invasive measurement of macroscopic plant’s parameters related to growth, architecture features or to main tissue components (Fiorani and Schurr, 2013). These systems are typically based on RGB color cameras to compute leaf area, biomass volume or to count/quantify specific plant organs, but they can also include the use of VIS-NIR hyperspectral cameras, to estimate the levels of main tissue components as chlorophylls, anthocyanins, and water, of fluorescence imaging devices, to map the efficiency of photosystems, or of thermal infrared cameras, to evaluate foliage temperature and leaf water status (Li et al., 2014; Fahlgren et al., 2015).

Early applications of color imaging to monitor lettuce growth were aimed to investigate the possible use of sensed plant-projected area extracted from top-view images to identify nutrient stress in hydroponic cultivation (Giacomelli et al., 1998). More recently, similar approaches have been applied to lettuce growth-rate data extracted from greenhouse imaging, to be used as state-variable to feedback control of nutrient solution in hydroponic system (Jung et al., 2015) or for other crop management operations (Kim et al., 2013). Bumgarner et al. (2012), by conducting an extensive study on imaging of lettuce plants grown in different environments, concluded that a top-view approach is an accurate method to indirectly measure lettuce biomass during the early stages of growth, while on canopy closure the correlation is weakened by occlusions in plant’s top-view due to leaves overlapping. This limitation, also reported by Jung et al. (2015) for lettuce plants at advanced stages of development, is encountered with any plant with erectophile architecture (Stewart et al., 2007; Tackenberg, 2007), and it has been addressed by deploying different approaches, as: by side-view imaging configurations, or, rarely, in combination with top-view (Pereyra-Irujo et al., 2012); by the use of three-dimensional (3D) measuring instrumentation such as LIDAR (Friedli et al., 2016), stereoscopic or multi-view cameras (Rose et al., 2015; Golbach et al., 2016) or time-of-flight (ToF) cameras (Chéné et al., 2012).

Low-cost 3D imaging sensors are emerging as an alternative to expensive 3D measurement systems, especially interesting for experiments involving small-scale, custom-made phenotyping hardware (Azzari et al., 2013; Paulus et al., 2014). The Microsoft Kinect V1 is a popular example of such a device, able to acquire at real time rate (30 frames per second) RGB color images aligned and synchronized with a depth D images. It can operate under indoor (or protected) illumination conditions, in a recommended distance range of 1–3.5 m, with a nominal depth error of ±10 mm (Livingston et al., 2012). A phenomic approach has recently been described in a study dealing with the evaluation of the efficacy of the biostimulant Megafol^®^ in reducing drought-stress related damage in tomato plants (Petrozza et al., 2014). Recently, an innovative method of selection and characterization of plant biostimulant matrices, involving a combination of technology, processes, and know-how, has been proposed (Povero et al., 2016).

Borage (*Borago officinalis* L.; Boraginaceae) is native to the Mediterranean region (Baubaire and Simon, 1987). The beneficial properties of chemicals extracted from different organs of this plant are widely acknowledged (for a review, see Asadi-Samani et al., 2014), and they are used in traditional medicine (Krishnaiah et al., 2011; Asadi-Samani et al., 2014), food preservation (Ciriano et al., 2009; Aliakbarlu and Tajik, 2012), and even for packaging purposes (Gómez-Estaca et al., 2009). The antioxidant properties of borage extracts from defatted seeds, leaves or flowers can mainly be ascribed to the presence of phenolic compounds (Wettasinghe et al., 2001; Aliakbarlu and Tajik, 2012). Borage leaves, that represent more than 60% of the plant matter, are considered also a low-cost crop by-product by some food processing industries (Garcia-Herreros et al., 2010).

On these premises, it appeared worthwhile to explore the possibility of using borage as a cheap source of biostimulants. Aim of the present work was to study the efficacy on lettuce plants (*Lactuca sativa* ‘Longifolia’) of foliar treatments with raw aqueous extracts obtained from leaves or flowers of *Borago officinalis* L. For this reason, a holistic approach has been adopted, including both traditional and innovative investigation techniques. Within the framework of this multidisciplinary study, a non-invasive measurement setup based on Kinect devices was implemented to evaluate plant growth (biomass) during time. Leaf functionality and stress responses were monitored by non-destructive measurements of chlorophyll *a* fluorescence, gas exchanges and by ethylene determination. Quality parameters such as concentrations of sugars, nitrates, and photosynthetic pigments, together with those of representative phenylpropanoid compounds (total phenols, flavonoids) and PAL activity and PAL-like polypeptide levels, were assessed by traditional biochemical methodologies. Eventually, a preliminary trial was set up in order to observe the effects of borage extracts integrated into packing films during storage of lettuce leaves.

## Materials and Methods

### Preparation and Chemical Characterization of Borage Extracts

Borage plants were harvested in the flowering stage in open field in Lodi province during spring (April). Borage flowers or leaves were minced, macerated in deionized water (500 g in 1 L) for 25 days, in the dark, at room temperature (RT). The aqueous extracts were filtered and properly diluted in water (to 1 or 10 mL L^–1^) to be used for treatments. In this preliminary work no surfactant was used, since we have not seen any problem in dispersion of foliar spray on the lettuce leaf surface. For chemical characterization, borage extracts were digested in wet conditions (0.1 M HNO_3_) and P, K, Ca, Fe, and Mn levels were measured by inductively coupled plasma mass spectrometry (ICP-MS; Bruker Aurora M90; Giro and Ferrante, 2016). Total N was determined with the Dumas method by using an elemental analyzer (ThermoQuest NA 1500 N; Thermo Electron, Milan, Italy). Total phenolic compounds in borage extracts were determined by the Folin–Ciocalteu’s procedure (Singleton et al., 1999; Kang and Saltveit, 2002). A 100 μL aliquot of extracts was diluted with 3.90 mL of double-distilled water and combined with 250 μL of 50% (v/v) Folin–Ciocalteu’s reagent and 750 μL of saturated (20% w/v) Na_2_CO_3_. Samples were vigorously shaken and incubated for 2 h, at RT in the dark before absorbance measurement at 765 nm. Total phenolics were expressed as gallic acid equivalents (GAE; mg L^–1^) upon comparison with a freshly prepared gallic acid standard curve. The pH values of aqueous extracts were measured by a Crison pH-Meter GLP 21^+^. The electrical conductivity was determined using a conductivity meter (Delta Ohm, Padova, Italy). Chemical characterization of extracts is reported as Supplementary Table S1.

### Plant Material and Treatments

Romaine lettuce (*Lactuca sativa* ‘Longifolia’) was obtained from a local nursery. Two-week-old plantlets were transplanted in 10 cm diameter plastic pots (nine pots per treatment), on a peaty substrate, in a greenhouse at the Faculty of Agricultural and Food Sciences of Milan, under controlled conditions. Environmental conditions in greenhouse during the experimental period were in average 20.3°C and 67% relative humidity. Treatment solutions were sprayed in the morning (between 09:00 and 10:00) onto lettuce leaves until run-off, at half cycle (13 days after transplanting) and 1 day before harvest (21 days after transplanting). The treatment conditions were: water (control plants); 1 or 10 mL L^–1^ of borage leaf extracts (LE); 1 or 10 mL L^–1^ of borage flower extracts (FE). Lettuce was harvested at commercial maturity stage. At harvest, after discarding the wrapper leaves from the lettuce heads, the next three non-injured leaves from four heads per treatment were carefully removed and 12 cm × 10 cm midrib sections were excised, starting at ca. 7 cm from the basis of the leaf. The pooled leaf sections from each plant were gently rinsed with distilled water, blotted with paper towels, immediately frozen in liquid N_2_, and stored at -80°C or at -20°C until use for biochemical analyses.

### Non-destructive Determinations

During the growth cycle and at harvest non-destructive analyses were conducted on fresh leaf tissue.

#### Estimation of Plant Growth

To evaluate the lettuce head biomass during time, an *in vivo* measurement technique was applied, consisting in acquiring and processing images from multi-angle side views of undisturbed potted lettuce plants. Images were acquired with Kinect V1 (Microsoft, United States). Measurements of lettuce head volume were conducted in a 1.3 m × 1.3 m × 1.8 m controlled-light cabinet where two Kinect V1 units were installed, one acquiring images from top and one from side view. A motorized table holder rotated the potted plant around its vertical axis during imaging, enabling to acquire 11 side images of each lettuce head viewed at angle steps of 30°. Top-view was aimed to monitor biomass growth during the very early stages of plant development. Since for this experiment the quantitative analysis of the growth was focused on plants at advanced development stages, only the measurements from the side-view imaging device were considered, thanks to the superior accuracy (i.e., reduced sensitivity on leaf occlusions) of this setup for more advanced growth stages. To this aim, the head-projected area was automatically segmented from the background of the cabinet in each of the 11 images, and the head volume computed by composing the side areas into a solid of revolution around the vertical axis of the plant. Lettuce image acquisitions were repeated at three different time points at 5-day intervals approximately, i.e., at dates corresponding to: 2 days before treatment 1, 3 days after treatment 1, and the same day of treatment 2. From the computed head volume (Vh; cm^3^) for each plant and each time point, an estimate of the corresponding fresh weight (FWh; g) was obtained through a linear model FWh = a0 + a1 × Vh. This equation was calibrated using a dataset collected in a complementary experiment, separately conducted on 78 lettuce plants grown in pots according to the control protocol. After transplanting, every fourth day a subset of six plants was imaged and destructively harvested to measure the FWh. A wide-range (from 2.5 to 155.8 g) set of 78 known values of FW and their corresponding values of computed volume was obtained. From a regression analysis computed with the Matlab 8.4 software package (MathWorks, Natick, MA, United States), the coefficients a0 = -1.97 g and a1 = 0.013 g × cm^-3^ were obtained with a root-mean-square error of calibration (RMSEC) of 2.2 g, to be used in the linear equation for non-invasive estimation of the lettuce heads biomass during growth by means of multi-angle side-view imaging of potted plants.

#### Chlorophyll *a* Fluorescence and Gas Exchange

Chlorophyll *a* fluorescence was measured 1 day after each treatment (i.e., 14 and 21 days after transplanting, respectively) using a hand-portable fluorometer (Handy PEA, Hansatech, Kings Lynn, United Kingdom). Leaves were dark-adapted for 30 min. Using a leaf clip (4 mm diameter), a rapid pulse of high-intensity light of 3000 μmol m^-2^ s^–1^ (600 W m^-2^) was administered to the leaf inducing fluorescence. The fluorescence parameters were calculated automatically by the used device. Leaf gas exchange rates were measured using the portable infrared gas exchange system CIRAS-1 (PP Systems, Hitchin, United Kingdom), operated in open-configuration with controlled temperature, CO_2_ concentration, and vapor pressure. Measurements were carried out on a fully expanded leaf between 09:00 and 13:00 h IT time. In the cuvette, during the recording time, light intensity was fixed to 1000 μmol^⋅^m^-2⋅^s^–1^ and CO_2_ concentration was set to 350 ppm.

### Destructive Determinations

#### Ethylene Emission

Whole lettuce heads were harvested the day after the second treatment. Each plant was enclosed in a 1.7 L airtight jar at 20°C. Ethylene was determined by withdrawing with a syringe, 3 h after jar sealing, a 1-mL headspace gas sample and injecting it into a Dani 3800 gas chromatograph (DANI Instruments S.p.A., Cologno M.se, Milan, Italy) equipped with a stainless steel column (100 cm long; 0.32 cm diameter) filled with Porapak Q at 100°C and a flame-ionization detector at 210°C. The carrier gas was N_2_ at 0.8 bar.

#### Chlorophylls and Carotenoids

Chlorophylls and carotenoids were determined in lettuce leaf tissue at harvest or after 7 days of storage in plastic bags. Leaf tissue (30–50 mg) was extracted using 100% (v/v) methanol, for 24 h at 4°C in a dark room; afterward quantitative determination of chlorophylls was carried out. Absorbance readings were measured at 665.2 and 652.4 nm for chlorophylls and 470 nm for total carotenoids. Pigment levels were calculated by Lichtenthaler’s formula (Lichtenthaler, 1987) and expressed on the basis of fresh weight of the tissue.

#### Nitrate

Nitrate concentration was measured by the salicylsulfuric acid method (Cataldo et al., 1975). One gram of fresh leaf tissue was homogenized (mortar and pestle) in 3 mL of distilled water. The extract was centrifuged at 3000 × *g* for 15 min at RT (ALC centrifuge-model PK130R) and the recovered supernatant was used for the colorimetric determination. Twenty microliters of sample were added to 80 μL of 5% (w/v) salicylic acid dissolved in H_2_SO_4_ plus 3 mL of 1.5 N NaOH. The samples were cooled at RT and absorbance at 410 nm was measured. Nitrate concentration was calculated referring to a KNO_3_ standard calibration curve.

#### Sugars

About 1 g of leaf tissue was homogenized in 3 mL of distilled water and centrifuged at 3000 × *g* for 15 min at RT. Sucrose and total sugars were assayed according to the resorcinol method and anthrone assay, respectively (Cocetta et al., 2015). Absorbance was read at 500 nm for sucrose and at 620 nm for total sugars and the levels were calculated referring to sucrose or glucose calibration curves, respectively.

#### Total Antioxidant Capacity

One gram of the frozen pooled leaf tissue was ground (mortar and pestle) in the presence of liquid N_2_ to a fine powder. Two volumes of 100% (v/v) methanol were added and the suspension was homogenized and centrifuged in a Sorvall RC-5B refrigerated centrifuge (10000 × *g*, 20 min, 4°C). The supernatant was recovered and the resulting pellet, resuspended in 0.5 mL of 70% (v/v) methanol, was centrifuged again. The two pooled supernatants were kept at 4°C until immediate use for spectrophotometric determinations. An aliquot of 0.1 mL of methanolic lettuce LE was combined with 1 mL of reagent solution [0.6 M H_2_SO_4_, 28 mM NaH_2_PO_4_, 4 mM (NH_4_)_6_Mo_7_O_24_], and incubated at 95°C for 90 min. After cooling to RT, the absorbance of the samples was measured at 695 nm in a UV-vis spectrophotometer (Secomam UviLine 9400). The levels of ascorbic acid-like substances were calculated from a standard curve obtained with a 10–150 μM freshly prepared ascorbic acid standard solution in 70% (v/v) methanol and extracts antioxidant capacity was expressed as ascorbic acid equivalents g^–1^ FW (AAE; Prieto et al., 1999).

#### Phenolic Compounds

Total phenolic compounds were assayed in the methanolic LEs by the Folin–Ciocalteu’s procedure as described in the paragraph “Preparation and Chemical Characterization of Borage Extracts,” and expressed as GAE (mg g^–1^ FW of the tissue) upon comparison with a standard curve obtained with freshly prepared gallic acid in 70% (v/v) methanol.

#### Total Flavonoids

Total flavonoids were determined according to Floegel et al. (2011). An aliquot of 500 μL of leaf methanolic extracts or standard solution (freshly prepared rutin dissolved in 70% v/v methanol) were mixed with 3.2 mL of double-distilled water. One-hundred and fifty microliters of 5% (w/v) NaNO_2_ solution were added and mixed, followed, after 5 min, by the addition of 150 μL of 10% (w/v) AlCl_3_. After 6 min, 1 mL of 1 M NaOH was added and absorbance at 510 nm of the colored flavonoid–aluminum complex was measured immediately. Total flavonoid concentration was expressed as nmol rutin equivalents g^–1^ FW of the sample.

#### PAL Extraction and *In Vitro* Activity Assay

Extraction and *in vitro* assay of PAL activity were conducted as described by Chen et al. (2006) and Jhin and Hwang (2015) with slight modifications. Two grams of frozen leaf tissue were homogenized (mortar and pestle) in the presence of liquid N_2_ with four volumes of a buffer containing 100 mM Tris-HCl (pH 8.8), 2 mM Na_2_-EDTA, 5 mM ascorbic acid, 1 mM PMSF, 5 mM MSH, 10% (w/w) PVPP. The samples were filtered through four layers of cheesecloth and centrifuged (15000 × *g*, 30 min, 4°C; Sorvall RC-5B); the supernatants, containing total soluble proteins, were used as crude enzyme extracts. The *in vitro* assay of PAL activity was conducted in a mix (1 mL total volume) containing 100 mM Tris-HCl buffer (pH 8.8), 20 mM (final concentration) phenylalanine and aliquots (100 and 200 μL) of crude enzyme extract, added to start the reaction. The mix was incubated at 38°C and the reaction stopped, after 0 min (blank), 30 and 60 min, by addition of 250 μL of 6 N HCl. After centrifugation, the absorbance at 290 nm of the recovered supernatants was read. One unit of PAL activity was defined as the amount of enzyme causing an increase of 0.01 in absorbance at 290 nm, equal to 3.09 nmol of CA formed per hour. PAL specific activity was then expressed on the basis of the tissue soluble protein concentration, determined by the Bradford method (Bradford, 1976) using bovine serum albumin as a standard (Micro-Bio-Rad Protein Assay; Bio-Rad Laboratories, Segrate, Italy).

#### SDS-PAGE and Western Blotting

Proteins denatured in sodium dodecyl sulfate (SDS) sample buffer (Laemmli, 1970) were analyzed by tricine-SDS-PAGE (10% total acrylamide/bis-acrylamide concentration; Schägger and von Jagow, 1987) in a Mini-Protean^TM^ apparatus (Bio-Rad Laboratories); gels were stained with Coomassie Blue R-250. Molecular weight markers were from Bio-Rad (Kaleidoscope Pre-Stained Standards).

Proteins were electro-blotted onto nitrocellulose membrane (0.2 μm, Amersham Life Science) in a Multiphor II Nova-Blot (Amersham Biosciences, Milan, Italy) apparatus (Morgutti et al., 2006). Protein transfer was carried out at RT at 0.8 mA cm^-2^. The membrane was blocked for 2 h in 3% (w/v) defatted milk in Tris-buffered saline-Tween buffer [TBS-T: 20 mM Tris-HCl (pH 7.6), 200 mM NaCl and 0.05% (w/v) Tween-20] and incubated overnight at 4°C with parsley anti-PAL polyclonal antisera (Dr. Imre E. Somssich) diluted (1:3000) in TBS-T. Blots, thoroughly washed with TBS-T, were incubated (2 h, RT) with alkaline phosphatase-conjugated anti-rabbit IgG from goat (Sigma; 1:30000 dilution). The membrane was stained with 10 mL of 5-bromo-4-chloro-3-indolyl phosphate/nitro blue tetrazolium (BCIP^®^/NBT; SIGMAFAST^TM^ tablets, Sigma–Aldrich).

### Preparation of Coated Plastic Bags and Postharvest Storage of Lettuce

Pullulan (PI-20 grade, Mw ∼200 kDa), an exopolysaccharide (EPS) produced by the yeast-like forms of the fungus *Aureobasidium pullulans*, was purchased from Hayashibara Biochemical Laboratories Inc. (Okayama, Japan). Oriented polypropylene (OPP, 30 μm), kindly provided by Bonduelle Srl (Milan, Italy), was used as a plastic substrate for the deposition of the active coating. Two different active coating solutions were prepared using the borage LEs and FEs, respectively. In both cases, a 10% (w/v) water solution was prepared. Six different pullulan solutions were prepared in water (10 wt.%, wet basis) under gentle stirring for 15 min at 25°C. Before coating deposition, the OPP films were treated with a high-frequency corona treatment (Arcotec, Mönsheim, Germany) to increase the film surface energy, improving plastic substrate-coating adhesion. An aliquot (∼5 mL) of the active solution was placed on the corona-treated side of the OPP film. The deposition of the coating solution was carried out by an automatic film applicator (ref 1137, Sheen Instruments, Kingston, United Kingdom), at a constant speed of 150 mm min^–1^ (ASTM D823-07-Practice C), using a horizontal steel rod with an engraved pattern, which yielded final coatings with comparable nominal thickness (1.0 μm) after water evaporation. Drying was performed using a constant and perpendicular air flux at 25.0 ± 0.3°C for 2 min at a 40 cm-distance from the applicator. Packaging of the lettuce leaves (about 20–25 g) was carried out using a Polikrimper TX/08 thermal heat sealer (Alipack, Pontecurone, Italy: 130°C; dwell time: 0.5 s; 4.0 bar) equipped with smooth plates. The postharvest trials were conducted by storing lettuce leaves up to 7 days at 4°C. Samples and conditions of packaging were: (A) control leaves packed in uncoated plastic bag; (B) control leaves packed in LE-coated plastic bag; (C) control leaves packed in FE-coated plastic bag; (D) 10 mL L^–1^ LE-treated leaves packed in uncoated plastic bag; (E) 10 mL L^–1^ FE-treated leaves packed in uncoated plastic bag. Analyses of total chlorophylls and carotenoids were performed at the end of the storage time period (*t* = 7 days) and compared to the levels measured at harvest (*t* = 0).

### Statistical Analysis

All data were subjected to one-way ANOVA and differences among means were determined by Bonferroni’s post test. Data referred to chlorophyll *a* fluorescence parameters and to postharvest trial were subjected to two-way ANOVA. Additional information is reported in the figure legends.

## Results

### Plant Growth

Growth (fresh weight) of lettuce plants treated (LE or FE, at 1 mL L^–1^ or 10 mL L^–1^ each) or not with borage extracts, was measured (data not shown). The average fresh weight of the control plant heads at harvest (22 days after transplanting) was 55.9 g. All treatments enhanced growth, with a maximum effect (+16%) at the highest LE and FE concentration. The stimulating effect was minimum (+6.44%) at 1 mL L^–1^ FE.

**FIGURE 1:**
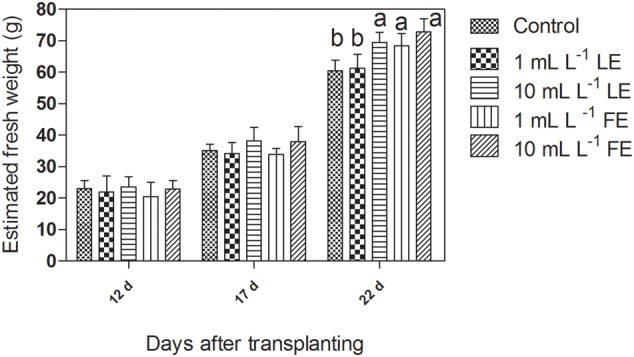
Estimated fresh weight of Romaine lettuce plants treated with water (control), 1 or 10 mL L^–1^ borage leaf (LE) or flower extract (FE). Data were obtained by processing of multi-view angles images from undisturbed potted lettuce plants at three time points of growth (days after transplanting). Values are means ± SE (*n* = 9). Data were subjected to one-way ANOVA. Different letters, where present, indicate significant differences among treatments.

**Figure 1** shows an overview of biomass growth during time, as estimated from the head volume computed from multi-view images through the introduced regression linear equation. For the three considered time points (12, 17, 22 days after transplanting), the mean value of estimated head plant fresh weight per each treatment is shown. Twelve days after transplanting, i.e., prior to any treatment, the estimated head mass ranged around 20–24 g, with no significant difference among groups, as expected. Seventeen days after transplanting, i.e., 4 days after the first treatment, the estimated average head weight (34 g) of plants treated with 1 mL L^–1^ of both LE and FE did not deviate from the control average (35 g), whereas some difference in the growth rate (average head weight 38 g) appeared in the groups treated with both LE and FE at the highest concentration (10 mL L^–1^). Nevertheless, the ANOVA did not reveal a significant (*P* < 0.05) difference between groups. After the second treatment (22 days after transplanting), i.e., just prior to harvest, the estimated head mass was significantly (*P* < 0.01) affected by both LE and FE treatments at 10 mL L^–1^ (+15% and +18%, respectively, compared to the control). Similarly, the 1 mL L^–1^ FE treatment exerted a significant (*P* < 0.01), albeit lower, stimulating effect (+13% over control). One mL L^–1^ LE exerted a very low stimulating effect (+4%). The values of head weight estimated from multi-view images at 22 days after transplanting were fairly related (*R*^2^= 0.74) to those of fresh weight measured immediately after harvest, even if with a relevant bias which led to a tendency to overestimate the absolute value of plant fresh weight.

#### Chlorophyll *a* Fluorescence

**FIGURE 2:**
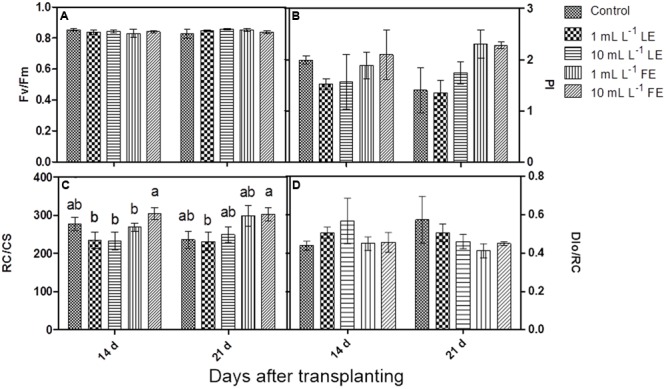
Chlorophyll *a* fluorescence parameters measured in Romaine lettuce plants treated with water (control), 1 or 10 mL L^–1^ borage LE or FE. **(A)** Maximum quantum efficiency of PSII, **(B)** performance index, **(C)** number of reaction centers per cross section, **(D)** energy dissipated per reaction center. Values are means ± SE (*n* = 3). Data were subjected to two-way ANOVA. Different letters, where present, indicate significant differences among treatments or times.

The maximum quantum efficiency of PSII (*F*v/*F*m) (**Figure 2A**) did not show any significant change in response to treatments; all samples yielded values higher than 0.83, commonly referred to as the threshold value between stressed and unstressed leaf tissue.

After the first treatment with borage extracts, the performance index (PI) did not show any significant change, even if the values were slightly lower in LE-treated plants, whereas FE-treated plants did not show any difference in comparison to the controls. After the second treatment, FE-treated samples showed a marked, even if not significant, increase in PI compared to controls and to LE-treated plants (**Figure 2B**). The positive effect of FE was confirmed by the higher number of reaction centers per cross section (RC/CS); in fact, the value of this parameter was significantly higher in FE-treated (10 mL L^–1^) plants than in the controls already after the first treatment. The second treatment induced a more evident effect: FE-treated samples showed significantly higher values of RC/CS compared to both controls and LE-treated ones (**Figure 2C**). Furthermore, the rate of energy dissipated by the PSII per reaction center (DIo/RC) was slightly lower in FE-treated plants compared to controls or LE-treated ones (**Figure 2D**).

#### Gas Exchange Measurements

**FIGURE 3:**
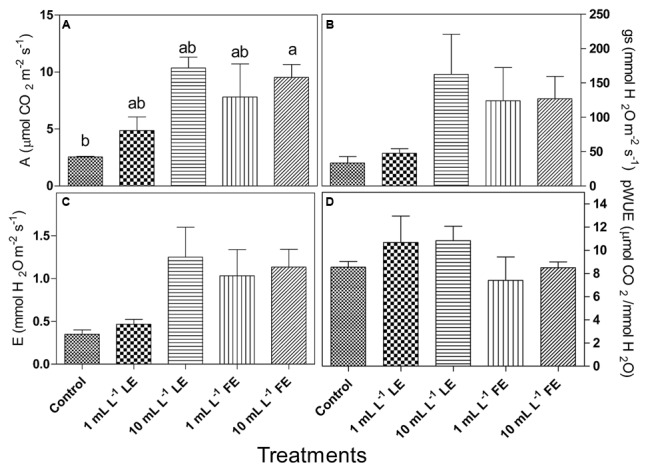
Leaf gas exchanges in Romaine lettuce plants treated with water (control), 1 or 10 mL L^–1^ borage LE or FE. **(A)** Net photosynthesis, **(B)** transpiration, **(C)** stomata conductance, **(D)** photosynthetic water use efficiency. Values are means ± SE (*n* = 3). Data were subjected to one-way ANOVA. Different letters, where present, indicate significant differences among treatments.

The considered parameters in this trial were net photosynthesis (*A*), stomata conductance (*g*s), transpiration (*E*), photosynthetic water use efficiency (pWUE) and intrinsic water use efficiency (iWUE). All treatments with borage extracts enhanced net photosynthesis, even if significant differences were only observed between controls and 10 mL L^–1^ FE-treated plants (**Figure 3A**). The effects of borage extracts on *g*s and *E*-values showed a similar trend (**Figures 3B,C**) even if no significant difference among treatments could be observed. Similar results were found for the pWUE (**Figure 3D**) and iWUE (data not shown) indexes.

#### Plant Ethylene Production

**FIGURE 4:**
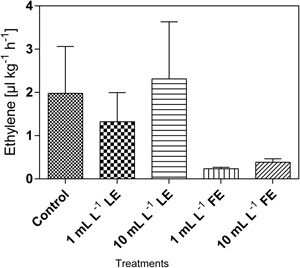
Ethylene emission in Romaine lettuce heads treated with water (control), 1 or 10 mL L^–1^ borage LE or FE. Values are means ± SE (*n* = 3). Data were subjected to one-way ANOVA.

The amount of hormone produced by both control and treated plants did not exceed 2.5 μL kg^-1^ h^-1^ (**Figure 4**). Lower amounts of ethylene production were recorded after FE treatment, irrespective of the applied dose. Ethylene produced in both controls and 10 mL L^–1^ LE-treated plants was by about 9- to 10-fold greater than that produced in 1 mL L^–1^ FE-treated plants. However, the effects were not statistically relevant due to high data variability.

#### Total Chlorophylls and Carotenoids

**FIGURE 5:**
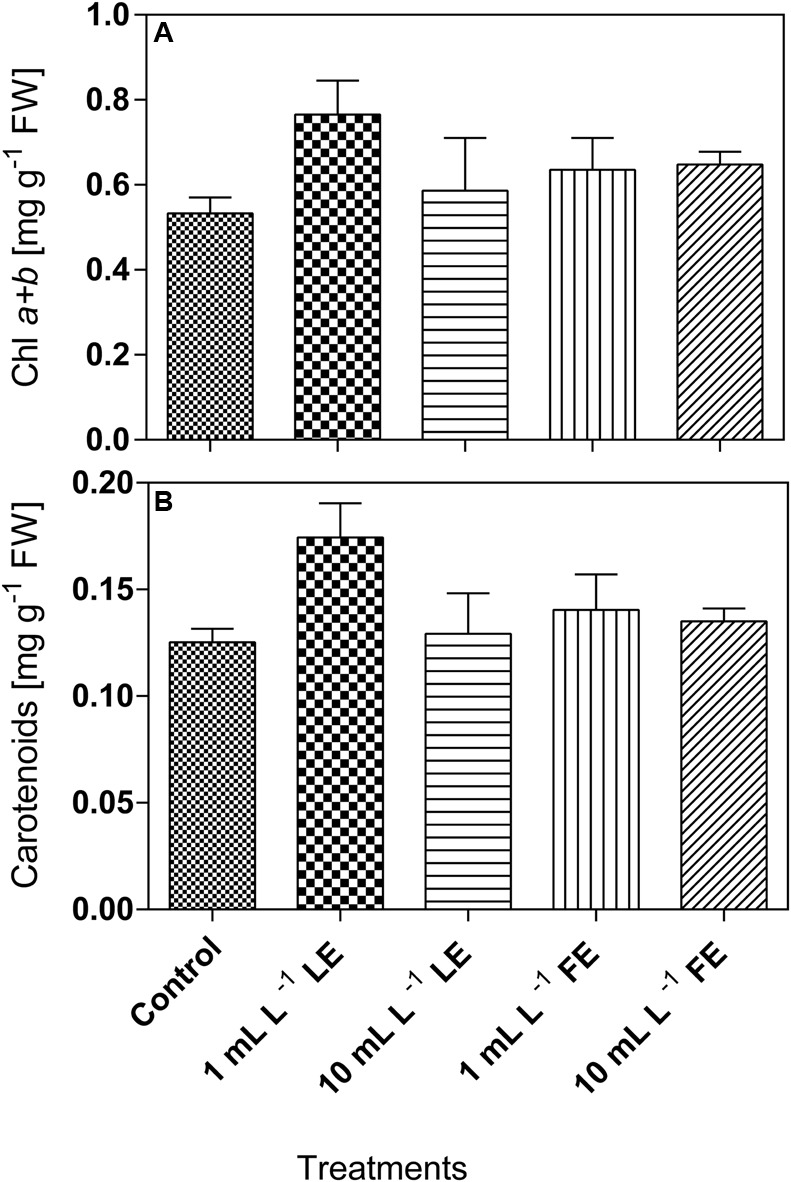
Chlorophyll *a*+*b* **(A)** and carotenoids **(B)** concentrations in Romaine lettuce leaf tissue treated with water (control), 1 or 10 mL L^–1^ borage LE or FE. Values are means ± SE (*n* = 3). Data were subjected to one-way ANOVA.

Lettuce leaf tissue treated with 1 mL L^–1^ LE showed the highest concentration of chlorophyll *a*+*b* (0.765 mg g^–1^ FW), and the same effect was observed for carotenoids (0.174 mg g^–1^ FW). In all cases, the concentrations of these pigments in plants treated with the borage extracts were slightly higher, even if not significantly different, than in the controls (**Figures 5A,B**).

#### Nitrate

**Table 1** shows the nitrate concentration in lettuce leaves treated or not with borage extracts. The absolute values of nitrate ranged from 138.9 to 236.2 mg kg^–1^ FW. LE-treated plants showed nitrate levels similar to those of the controls, whereas the FE-treated ones showed slightly higher nitrate levels.

**Table 1:** Nitrate and sugars concentrations of Romaine lettuce leaf tissue treated with water (control), 1 or 10 mL L^–1^ borage leaf (LE) or flower extract (FE).

	Nitrate [mg kg^–1^ FW]	Sucrose [mg kg^–1^ FW]	Total sugars [mg kg^–1^ FW]
Control	138.9 ± 12.9	1885.2 ± 316.5	2785.9 ± 476.9
1 mL L^–1^ LE	164.4 ± 13.9	1658.3 ± 72.3	2538.4 ± 405.2
10 mL L^–1^ LE	138.5 ± 8.70	1489.7 ± 203.5	1551.6 ± 218.4
1 mL L^–1^ FE	195.3 ± 49.7	1313.1 ± 160.6	2517.4 ± 52.6
10 mL L^–1^ FE	236.2 ± 13.9	1283.7 ± 200.0	1907.4 ± 486.1

*Values are means ± SE (*n* = 3). Data were subjected to one-way ANOVA.*

#### Sucrose and Total Sugars

The highest concentration of sucrose (**Table 1**) was found in leaves of control plants (1885.2 mg kg^–1^ FW), while borage extracts (and particularly so FE) induced a decrease in this parameter, even if the observed differences among treatments were not statistically significant. Also for total sugars, control plants showed the highest value (2785.9 mg kg^–1^ FW), and 10 mL L^–1^ LE induced a decrease in this parameter (1551.6 mg kg^–1^ FW; **Table 1**).

#### Total Phenols and Flavonoids and Total Antioxidant Capacity

**FIGURE 6:**
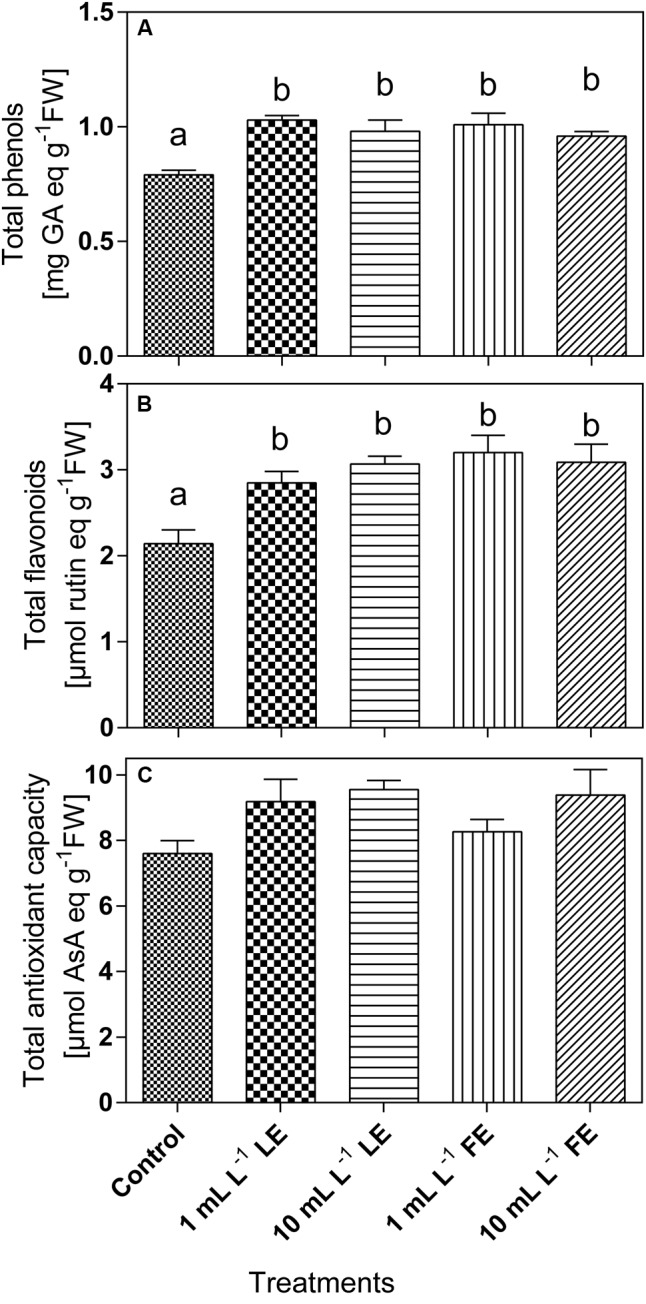
Phenolics **(A)** and total flavonoids **(B)** concentrations, and antioxidant capacity **(C)** in Romaine lettuce leaf tissue treated with water (control), 1 or 10 mL L^–1^ borage LE or FE. Values are means ± SE (*n* = 8). Data were subjected to one-way ANOVA. Different letters, where present, represent significant differences among treatments.

The phenolics concentration (**Figure 6A**) in the leaf tissue of control plants was 0.82 mg GAE g^–1^ FW and increased upon treatment with borage extracts. In particular, the values recorded were significantly increased by all treatments (+26.3%, +19.6%, +23.5%, +17.2% by 1 mL L^–1^ LE, 10 mL L^–1^ LE, 1 mL L^–1^ FE, and 10 mL L^–1^ FE, respectively). Also the concentrations of total flavonoids (**Figure 6B**) were increased upon treatments with borage extracts. In the leaves of control plants a value of 2.37 μmol rutin equivalents g^–1^ FW was observed, that was significantly increased by all treatments (+20.0%, +24.2%, +34.7%, +21.7% by 1 mL L^–1^ LE, 10 mL L^–1^ LE, 1 mL L^–1^ FE, and 10 mL L^–1^ FE, respectively). The antioxidant capacity was 8.01 AAE g^–1^ FW in the control plants, and it showed a general tendency to increase upon treatment with borage extracts (**Figure 6C**).

#### Total Soluble Proteins

**FIGURE 7:**
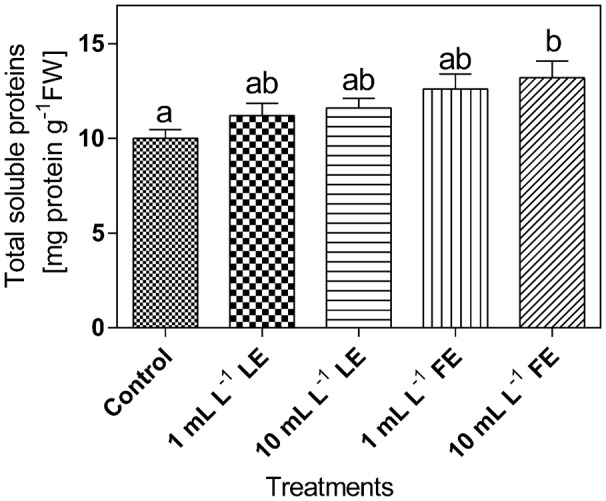
Total soluble proteins in Romaine lettuce leaf tissue treated with water (control), 1 or 10 mL L^–1^ borage LE or FE. Values are means ± SE (*n* = 8). Data were subjected to one-way ANOVA. Different letters represent significant differences among treatments.

The levels of total soluble proteins in lettuce leaf tissue (**Figure 7**) were affected by the treatments with borage extracts. In fact, the lowest amount of soluble proteins (∼10 mg g^-1^ FW) was observed in the control plants; increases of +12%, +16%, +26%, and +32% were induced by 1 mL L^–1^ LE, 10 mL L^–1^ LE, 1 mL L^–1^ FE, and 10 mL L^–1^ FE, respectively. In particular, the highest and significant effect was induced by the treatment with 10 mL L^–1^ FE.

#### *In Vitro* PAL Activity and PAL-like Polypeptide Levels

**FIGURE 8:**
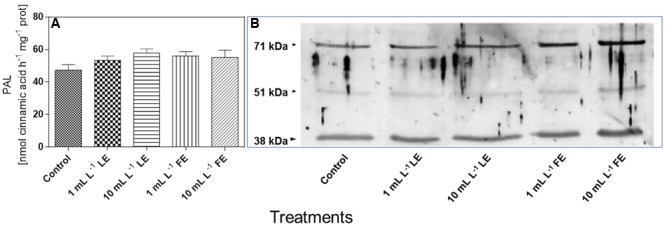
*In vitro* PAL specific activity **(A)** and levels of PAL-like polypeptides **(B)** in Romaine lettuce leaf tissue treated with water (control), 1 or 10 mL L^–1^ borage LE or FE. *In vitro* PAL activity data are means ± SE (*n* = 8). For immunoblotting, polyclonal antibodies raised against a PAL protein of *Petroselinum crispum*, (kind gift of Dr. Imre E. Somssich) were used. Loading was 10 μg protein per lane. The results of one experiment, representative of three, are shown.

**Figure 8A** shows that the *in vitro* PAL specific activity in leaves of the control plants was 47.3 nmol CA h^–1^ mg^–1^ soluble protein. All treatments with borage extracts enhanced, albeit not significantly, the enzyme activity, with an average effect for the four treatments of about +17%. The levels of PAL-like polypeptides were also assessed in the same soluble protein extracts used for the determination of *in vitro* PAL activity. **Figure 8B** shows that, in all soluble protein extracts of the lettuce leaves, the anti-PAL antibodies from parsley yielded a clear immunogenic signal against two polypeptides of approximately 71 and 38 kDa, reacting also, even if only weakly, with a polypeptide of approximately 51 kDa. The signal against the three PAL-like polypeptides showed a tendency to increase upon all four borage treatments, and particularly so in the case of FEs (1 and 10 mL L^–1^).

#### Effect of Borage Extracts during Storage

In general, a positive effect of borage extracts was observed on total chlorophylls and carotenoids concentrations during cold storage. In fact, leaves subjected to all kinds of treatment showed higher concentrations after 7 days at 4°C compared to those at harvest, even though the observed increments were not significant. At the end of the storage period the only significant increment of both chlorophylls and carotenoids was observed in FE-treated lettuce leaves packed in uncoated bags (**Table 2**). After cold storage, no apparent decay symptoms were recorded in any sample.

**Table 2:** Effects of borage leaf (LE) or flower extracts (FE), administered *in vivo* to lettuce plants or as coatings of plastic films, on total chlorophylls and carotenoids concentration of lettuce leaves at harvest (0 days) and after 7 days of storage at 4°C.

Storage time/coating type	*In vivo* plant treatment					
	Control	LE	FE	Control	LE	FE
	Chl *a*+*b* [mg g^–1^ FW]			Carotenoids [mg g^–1^ FW]		
0 day/-	0.53 ± 0.04ˆab	0.59 ± 0.12ˆab	0.65 ± 0.03ˆab	0.13 ± 0.01ˆab	0.13 ± 0.02ˆab	0.14 ± 0.01ˆab
7 days/-	0.48 ± 0.01ˆb	0.68 ± 0.24ˆab	0.97 ± 0.17ˆa	0.12 ± 0.02ˆab	0.17 ± 0.06ˆab	0.24 ± 0.04ˆa
7 days/LE	0.86 ± 0.24ˆab	–	–	0.22 ± 0.05ˆab	–	–
7 days/FE	0.84 ± 0.10ˆab	–	–	0.22 ± 0.02ˆab	–	–

*For the *in vivo* experiment, plants were treated with water (control) or 10 mL L^-*1*^ borage extracts, as described in “Materials and Methods”; leaves were then packed in uncoated or LE- or FE-coated plastic bags. Values, subjected to two-way ANOVA, are means ± SE (*n* = 3). Different letters indicate significant differences between treatments.*

## Discussion

Biostimulants act at different levels, increasing plant growth, photosynthetic and metabolic activities and nutrient absorption (Bulgari et al., 2015; Yakhin et al., 2017). The production of a potential biostimulant begins with raw material characterization followed by the study of plant responses (Povero et al., 2016). The effect of raw material extracts has to be evaluated under normal or stress conditions by investigating the physiological and biochemical processes that are activated after treatments. Successful biostimulant candidates should increase biomass and yield or counteract the negative effect of different stresses. Their use is nowadays becoming a common practice in crop production to improve productivity and yield. In leafy vegetables, the biostimulant Actiwave^®^ (containing betaine, alginic acid, and caidrine) applied as an additional component to the nutrient solution increases yield of rocket grown in a floating system, even with reduced nutrient concentrations (Vernieri et al., 2005); its effect was confirmed when administered as a spray on baby leaf lettuce grown in plastic tunnel (Amanda et al., 2009). An extract of brown marine algae was reported to increase growth of spinach *in vitro* (Fan et al., 2013). Dudaš et al. (2016) observed, on winter production of lettuce ‘Four Seasons,’ that the plant head mass was by 30% higher after treatment with Bio-algeen S-90 compared to control plants. Sternecker and Balas (2014) observed in lettuce ‘Mathilda’ a head weight increase of 31% upon use of a biostimulant composed by a mixture of extracts from 21 plant species associated with *Lactobacillus* and yeast. The increment that we observed in Romaine lettuce growth is consistent, in spite of the high variability of results, with the cited literature reports, and supports the hypothesized role of borage extracts in stimulating the biomass of treated plants.

Imaging methods have been successfully used for non-invasive estimation of plant growth (Tackenberg, 2007) also after biostimulant treatments (Povero et al., 2016), but literature concerning their application to lettuce is scanty and relies upon a top-view imaging of plant heads. Due to leaf overlap after canopy closure, this approach revealed a generally weak correlation between image-based and destructive measurements of biomass, as growth progressed. For ‘Outredgeous,’ Bumgarner et al. (2012) observed a decrease in correlation coefficient from *r* = 0.87 at 10 days after sowing to *r* = 0.22 at harvest (28 days). Similarly, Jung et al. (2015) reported a sharp decrease in the correlation when lettuce heads have a fresh weight above 25 g, even if they found an overall RMSEC of less than 5 g when estimating the biomass of samples with fresh weight ranging up to 70 g. In this study, the adopted multi-angle, side view approach enabled to define a linear model capable to estimate the lettuce biomass through image data with a RMSEC = 2.2 g, for fresh weight values up to 155 g. This model was successfully applied to monitor non-invasively the growth of lettuce heads as affected by borage extracts application, and the multiple side view approach allowed capturing the subtle effects of the treatments during plant growth. The application of multi-angle, side view imaging, instead of classical top-view approach, allowed obtaining a fair correlation (*R*^2^= 0.74) with the destructive harvest data even for plants at advanced growth (i.e., commercial harvest stage). It must be noted that the multi-angle approach used in this work can be successfully applied when conducting phenomic studies, but it does not appear suitable for on-the-go measurements in field or in greenhouse, where top-view imaging setup is the best option thanks to its much simpler implementation.

In lettuce, ethylene production is extremely low compared to other plant tissues (Burg, 2004). In lettuce ‘Acephala’ values of ethylene production lower than 10 μL kg^–1^ h^–1^ are reported (Diaz et al., 2007). Concerning Romaine lettuce, to our knowledge, only scanty literature is available about ethylene production. Regarding the effects of borage extracts described in the present work, it is interesting to notice that, despite the high variability of the results, possibly due to the extremely low levels of ethylene emission, in three out of the five experimental conditions a decrease in ethylene production was induced by borage extracts, particularly evident upon FEs administration, suggesting a healthier physiological status in the FE-treated plants. This result could be explained considering the antioxidant activity due to the presence of radical-scavenging components reported for crude *B. officinalis* extracts (Bandoniene and Murkovic, 2002; Bandoniene et al., 2005), that may play a role in counteracting the effects of potential stress factors and the related ethylene production.

Biostimulants enhance plant growth and total photosynthesis determining higher dry matter accumulation in vegetable and ornamental crops (Khan et al., 2009; Bulgari et al., 2015; Massa et al., 2016). Chlorophylls (also important for the visual appearance of the produce) and carotenoids (photoprotective molecules whose amount is related to that of chlorophyll) are involved in fundamental photochemical processes tightly associated with crop biomass production. Moreover, carotenoids and chlorophylls play an important role in preventing various human chronic-degenerative diseases associated with oxidative stress (Znidarcic et al., 2011), contributing to the nutraceutical quality of plant produce (Yuan et al., 2015). Biostimulant treatments are often able to increase leaf pigments concentration. In rocket, treatments with a *Moringa oleifera* extract increased chlorophyll and carotenoids levels (Abdalla, 2013); similar results were obtained with the biostimulant Actiwave^®^ (Vernieri et al., 2005). The commercial product ONE^®^ had positive, dose-dependent effects, on the chlorophylls levels of lettuce and endive (Bulgari et al., 2014). Consistently, borage extracts (in particular 1 mL L^–1^ LE) slightly increased the chlorophyll and carotenoids levels compared to controls.

Leaf functionality is also described by gas exchange analysis or estimated by chlorophyll *a* fluorescence. These non-destructive methods can be applied to evaluate the health status of the photosynthetic apparatus or the different responses of plant tissues to stress factors or experimental treatments (Murchie and Lawson, 2013). In the present work, a positive effect of borage FEs may be suggested by the higher values of PI, a general index of the leaf health status. Moreover, the higher number of reaction centers and lower rates of energy dissipation confirmed the hypothesis of a direct positive effect of the treatment on PSII efficiency. In lettuce, significant changes in the *F*v/*F*m ratio (a good indicator of leaf stress), usually observed after a mid- or long-term exposure to a specific treatment or stressful condition (Stepień and Kłbus, 2006), are considered an index of irreversible photoinhibition of PSII reaction centers (Dias et al., 2014). In our material, the *F*v/*F*m values did not show any significant change, suggesting a general positive effect of extracts on leaf functionality.

Biostimulant applications in coriander under cold stress were able to increase the *F*v/*F*m ratio, the transpiration, and stomatal conductance rates, but reduced intercellular carbon dioxide concentration (Pokluda et al., 2016), suggesting that biostimulants may accelerate the adaptation to chilling. The chlorophyll *a* fluorescence-derived parameters have been used for evaluating the vitality of transplant-sensitive tree species after transplanting, and the effects ohe effects of biostimulants application, that increased leaf functionality as shown by higher values of PI (Fraser and Percival, 2003).

Our results showed that the highest doses of both types of borage extracts increased the net photosynthesis as revealed by gas exchange analysis. In strawberry, Actiwave^®^ increased the photosynthetic activity by 27% compared with control (Spinelli et al., 2010). Consistent results were found in ornamental plants treated with a municipal biowaste: hibiscus plants showed an increase of net photosynthesis by 24% (Massa et al., 2016) and similar findings were observed in *Euphorbia* × *lomi* (Fascella et al., 2015).

Biostimulants improve the primary metabolism of plants, increasing the levels of free amino acids, proteins, carbohydrates, pigments, and various enzymes as reported by Yakhin et al. (2017).

In our material, the leaf sucrose levels were not affected by any borage extract treatment, suggesting that neither the nutritional nor the sensorial quality of the produce were significantly altered. However, the tissue levels of total sugars were diminished by all treatments, whereas the levels of some secondary metabolites, like total phenolics and flavonoids increased, as well as the antioxidant capacity. The opposite changes in the levels of total sugars and phenylpropanoid compounds would contribute to the health-related characteristics of the produce, at the same time maintaining a high level of chemical defense capability (Neilson et al., 2013) and, in turn, a better performance in terms of plant growth. A better general status of the plants treated with borage extracts, as well as potential higher resistance to stress factors for the presence of phenolic substances, is also suggested by the higher levels of total soluble proteins, indicative of the bulk of metabolic activity (Veerasamy et al., 2007 and references therein). The observed tendency to lower ethylene production is consistent with this view. Several primary metabolites (like free amino acids, sugars or other molecules not immediately required for growth and development) are precursors of secondary compounds, among which polyphenols (Mazid et al., 2011). In particular, the deamination of phenylalanine to trans-CA catalyzed by PAL links the primary metabolism to the production of a wide variety of secondary phenolic compounds, that serve diverse functions in plants, including protection against biotic and abiotic stresses, cellular signaling, mechanical support (MacDonald and D’Cunha, 2007). Our results on *in vitro* PAL activity and levels of PAL-like polypeptides are, in general, coherent with the results on total phenolics and flavonoids concentrations, even if we could not observe a tight correlation between the cited parameters. This result might be attributed to the very complex regulation of this enzyme, that involves several steps, from *PAL* (iso)genes transcription to assembling of the functional protein, and to enzyme turnover and mechanisms of activity regulation (phosphorylation–dephosphorylation); also feedback control by the levels of total phenolics/flavonoids is reported to regulate PAL protein turnover and catalytic activity (Zhang and Liu, 2015).

High dietary nitrate intake is hazardous for health, since in the human organism nitrate is reduced to nitrite that can react with the free amines deriving from protein digestion and form carcinogenic nitrosamines. For this reason, the European Union has posed limits in nitrate concentrations of commercialized leafy produce (Cavaiuolo and Ferrante, 2014). Nitrate accumulation in leafy vegetables is affected by several environmental factors like light intensity, photoperiod, and temperature (Lillo, 1994 and references therein). Biostimulants reduce the nitrate levels in several species of leafy vegetables (Vernieri et al., 2005; Liu and Lee, 2012; Dudaš et al., 2016). The borage treatments applied in the present work did not significantly affect nitrate in the lettuce cultivar used. Nitrate concentration shows considerable variations in different lettuce cultivars (from 26 mg kg^–1^ to more than 2500 mg kg^–1^; Cometti et al., 2011). In our material, the nitrate levels were lower than approximately 250 mg kg^–1^ FW, possibly explaining the observed lack of effect of borage treatments, similar to what observed for Actiwave^®^-treated baby leaf lettuce (Amanda et al., 2009). It should also be stressed that the effect of biostimulants on nitrate levels in leaves can be different depending on the species/cultivar and it is affected, in addition to environmental factors, by dose and time of application (Kunicki et al., 2010).

The effect of borage extracts, containing themselves bioactive molecules or possibly releasing volatile compounds (VOCs), during postharvest of lettuce leaves, was also evaluated in a preliminary trial. The visual appearance (chlorophyll) of the produce and leaf carotenoids levels, both known to be affected by storage conditions (Bolin and Huxsoll, 1991; Bergquist et al., 2007; Agüero et al., 2008) were assessed. Borage extracts, administered either as *in vivo* treatment to plants or applied as coating on packaging films, exerted a positive effect on the photosynthetic pigments, preventing their degradation, and even inducing their increase during storage. In particular, 10 mL L^–1^ FE proved capable to induce a significant increase in total chlorophylls and carotenoids after 7 days of storage as compared to the controls. However, further experiments will be necessary in order to investigate the mechanism of action of the bioactive compounds potentially present in borage extracts, when incorporated into a primary packaging. Moreover, a study of the release kinetics of such active molecules in the package headspace may also help to understand whether the effects of these treatments is linked to the production of active VOCs and/or is due to a direct contact between the produce and the inner surface of the packaging.

Taken as a whole, the multidisciplinary approach used in this work demonstrated that borage extracts do indeed exert biostimulant effects on lettuce plants. This result suggests a possible exploitation of borage extracts in vegetable production of different species, as well as in their commercialization, to improve quality and nutraceutical properties and thus adding value to produce. Moreover, the phenomic approach adopted proved capable to estimate with good accuracy plant growth and represented, in general, a fast and reliable method for the non-destructive screening of the efficacy of experimental treatments, integrating well the biochemical–physiological approach. Aspects related to both primary and secondary metabolism were enhanced, suggesting a potential ability of these extracts to counteract possible stress factors. In particular, FEs proved more effective than LEs on the plant physiological and biochemical parameters considered. These results may be validated at molecular level by studying the transcriptional profiles using high-throughput technological tools like microarrays and RNA-seq. The molecular mechanisms elicited by crude plant extracts acting as biostimulants were recently studied, in *Arabidopsis thaliana*, through a microarray-based genomic approach (Santaniello et al., 2013). A transcriptional profiling of phenylpropanoid pathway genes in *Arabidopsis thaliana* as affected by application of microbial products has been recently published (Ali and McNear, 2014). These additional research activities will also allow describing more completely the efficacy of borage extracts in preserving and enhance crop performance. Moreover, the effects of borage extracts and surfactants could be explored in future works in order to take into consideration other factors that can affect the effectiveness of foliar treatments (Fernandez and Brown, 2013).

## Conclusion

Our results appear suitable to be fruitfully included in a larger, integrated framework where different approaches are systematically combined (Povero et al., 2016) in order to study at different levels the potential positive effects of various natural extracts on plant performance and biochemical–physiological parameters related to qualitative features, eventually characterizing and validating these extracts as new biostimulant products exploitable in the field.

## Author Contributions

RB, substantial contribution to the experimental work, interpretation of data, and drafting of the manuscript; SM and NN, substantial contribution to the biochemical work, interpretation of data, drafting and critical revision of the manuscript; GC, elaboration and critical interpretation of data, drafting of the manuscript; SF designed and carried out the experiments on the coated plastic films; AC, EF, and RO, integration of the experimental setup for multi-view imaging of plants, imagery data acquisition and processing, and drafting of the manuscript; IM and AS carried out the ethylene production study and analysis of the data, and participated in the experimental design, critically reading the manuscript drafts; AF, experimental design and coordination of the work, interpretation of data, drafting and critical revision of the manuscript. All authors read and approved the final version of the manuscript.

## Conflict of Interest Statement

The authors declare that the research was conducted in the absence of any commercial or financial relationships that could be construed as a potential conflict of interest.
