# Supplementary material for: Evaluation of Borage Extracts As Potential Biostimulant Using a Phenomic, Agronomic, Physiological, and Biochemical Approach
Source: Front Plant Sci. 2017 Jun 7;8:935. doi: 10.3389/fpls.2017.00935 (PMC5461430; doi:10.3389/fpls.2017.00935)
Supplement: Supplementary file 1 [file Table_1.DOCX]

|  | P | K | Ca | Fe | Mn | N-NO_3_ | N-NH_4_ | PHENOLS | pH | EC |
| --- | --- | --- | --- | --- | --- | --- | --- | --- | --- | --- |
|  | (mg L^-1^) | (mg L^-1^) | (mg L^-1^) | (mg L^-1^) | (mg L^-1^) | (mg L^-1^) | (mg L^-1^) | (mg L^-1^ GAE) |  | (mS) |
| LE | 56762 | - | 190678 | 263 | 120 | 0.16 | 213 | 364 | 6.31 | 6.38 |
| FE | 17966 | - | 52322 | 399 | 70 | 0.29 | 88 | 242 | 9.23 | 4.31 |

Table s1. Chemical characterization of borage leaf (LE) and flower extract (FE).
